# Retrobulbar spot sign in non-ischemic conditions: a case series challenging its diagnostic role

**DOI:** 10.3389/fmed.2026.1764893

**Published:** 2026-05-20

**Authors:** Chuanjie Yin, Jiezhi Zhan, Xiliang He, Zhaohu Yu, Yu Sun, Shanshan Zhang, Huadong Lou

**Affiliations:** 1 Department of Ophthalmology, Shandong Second Medical University Affiliated Qingdao Eighth People's Hospital, Qingdao, Shandong, China; 2 Department of Ultrasound, Shandong Second Medical University Affiliated Qingdao Eighth People's Hospital, Qingdao, Shandong, China; 3 Department of Brain Center, Shandong Second Medical University Affiliated Qingdao Eighth People's Hospital, Qingdao, Shandong, China

**Keywords:** case report, central retinal artery occlusion, diagnosis, retrobulbar spot sign, ultrasonography

## Abstract

**Objectives:**

To present three cases where the retrobulbar spot sign (RBSS) was observed, but central retinal artery occlusion (CRAO) was not confirmed.

**Methods:**

Transorbital sonography was employed to detect the presence of RBSS in all patients.

**Results:**

RBSS was detected in all three patients. However, only one patient had CRAO, with RBSS-positive findings in both eyes, although one eye showed normal function. The other two patients exhibited RBSS without evidence of CRAO. RBSS remained unchanged in all four eyes during the follow-up period.

**Conclusion:**

RBSS may not be specific to CRAO, as it was observed in non-CRAO cases. Further research is necessary to clarify its diagnostic significance and differentiate it from other pathologies.

## Introduction

Central retinal artery occlusion (CRAO) is a critical ophthalmic emergency, commonly attributed to embolic events. In this diagnostic process, the “retrobulbar spot sign” (RBSS) detected by point-of-care ultrasound (POCUS) has been identified as a notable feature (1). RBSS was thought to indicate a plaque in the retrobulbar vessels, though direct pathological evidence remains limited. Traditionally, RBSS has been interpreted as evidence of a localized embolus obstructing the central retinal artery (CRA), contributing to disease assessment and prognosis. To date, RBSS has been observed exclusively in CRAO cases (2), with no studies investigating its presence or significance in other conditions. In this study, we present three eyes where RBSS was detected without any clinical signs or symptoms of CRAO. These findings suggest that RBSS may not be exclusive to CRAO, highlighting the need for further research into its potential diagnostic implications.

## Case presentation

**Figure 1:**
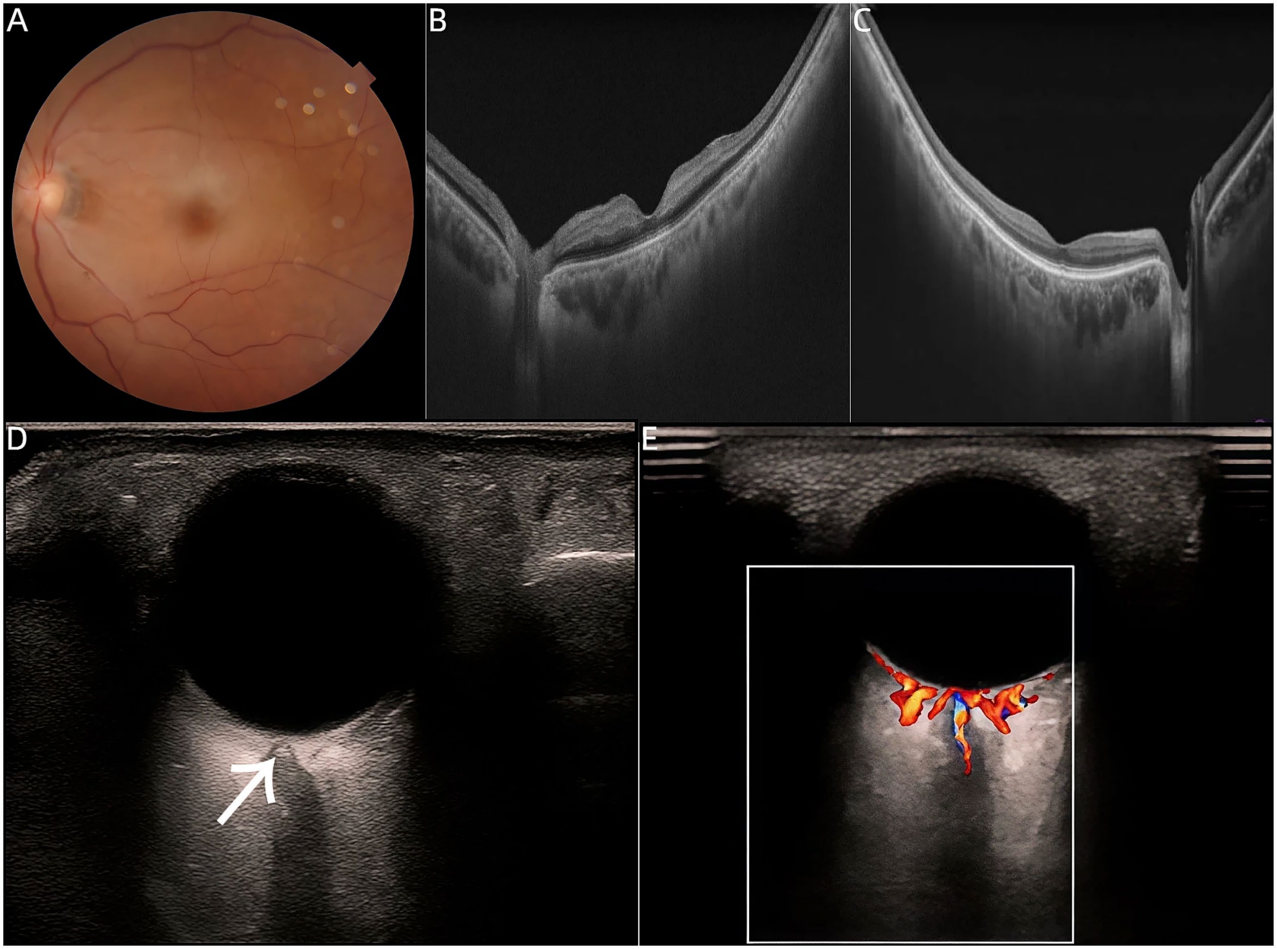
**(A)** Color fundus photography revealed perifoveal retinal whitening with a classic cherry-red spot at the foveola in the left eye. **(B)** OCT of the left eye demonstrated hyper-reflectivity confined to the inner nuclear layer. **(C)** OCT of the right eye showed normal retinal thickness and well-preserved laminar architecture across all retinal layers. **(D)** Ultrasound image showing a hyperechoic retrobulbar spot sign (RBSS) within the optic nerve in the right eye in Case 1 (arrow), measuring 1.4 × 1.3 mm. **(E)** Despite the presence of RBSS, ultrasound demonstrates abundant arterial blood flow in the right eye.

In the first case, an 80-year-old male presented to the clinic with a sudden decrease in vision in his left eye for the past 1.5 h. On ocular examination, his best-corrected visual acuity (BCVA) was 0.4 in the right eye and hand motion (HM) close to the eye in the left eye. Intraocular pressure (IOP) was normal in both eyes. Following comprehensive evaluations, including color fundus photography, optical coherence tomography (OCT), a diagnosis of nonarteritic CRAO in the left eye was confirmed (Figures 1A,B). With informed consent, intra-arterial thrombolysis for CRAO was performed. Briefly, 50 mg tissue plasminogen activator (rtPA) was used for thrombolysis. The procedure was successfully completed, resulting in an improvement of BCVA to finger counting at 30 cm (FC/30 cm). At the two-month follow-up, the patient’s left eye BCVA remained at FC/30 cm. Transorbital sonography demonstrated RBSS in both eyes, with more pronounced findings in the right eye (Figure 1D). Notably, the right eye had no history of visual loss and retained a BCVA of 0.4. OCT showed a normal retina (Figure 1C), and transorbital sonography revealed abundant arterial blood flow (Figure 1E). These findings provide the first evidence that RBSS can occur independently of an acute ischemic event.

**Figure 2:**
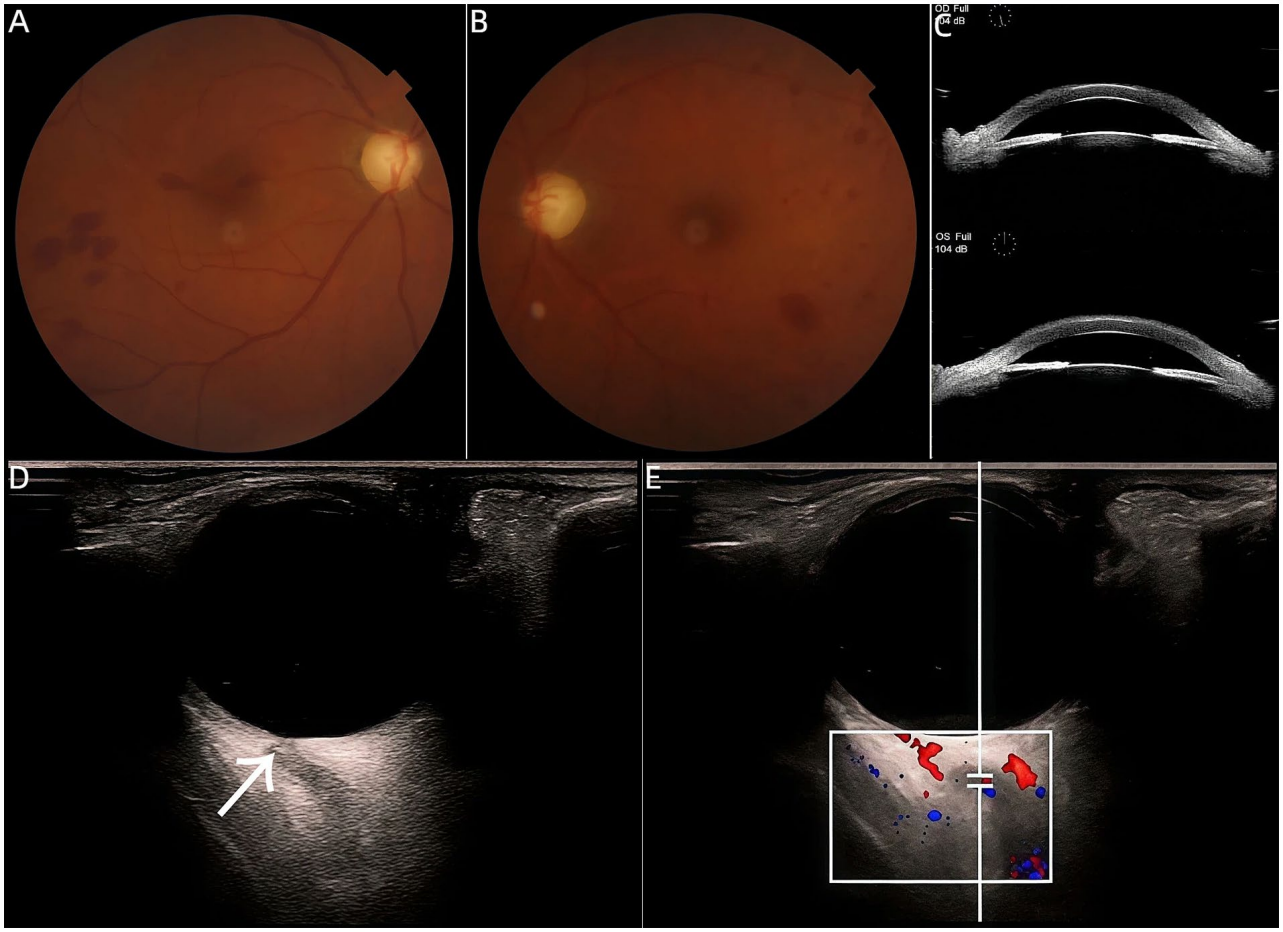
**(A,B)** Color fundus photography revealed a well-defined optic disc with a cup-to-disc (C/D) ratio of approximately 1.0 and multifocal intraretinal hemorrhages in both eyes. **(C)** UBM demonstrated a closed anterior chamber angle in both eyes. **(D)** Positive RBSS in Case 2 (arrow), measuring 1.0 × 0.8 mm. **(E)** Positive RBSS in the left eye, with arterial flow showing a peak systolic velocity (PS) of 8.19 cm/s and a resistive index (RI) of 0.54.

Secondly, a 45-year-old woman presented to the clinic with a 3-month history of bilateral vision loss. On ocular examination, her BCVA had no light perception (NLP) in both eyes. IOP was markedly elevated, measuring 59.3 mmHg in the right eye and 57.7 mmHg in the left eye. Bilateral corneal edema was noted, and the pupils were non-responsive to light. Color fundus photography revealed multifocal intraretinal hemorrhages in both eyes (Figures 2A,B). Ultrasound Biomicroscopy (UBM) demonstrated anterior synechiae of the iris and a closed anterior chamber angle (Figure 2C). A diagnosis of primary angle-closure glaucoma was made. B-scan ultrasonography demonstrated normal retrobulbar blood flow, with a positive RBSS in the left eye (Figure 2D) and near-normal arterial flow (Figure 2E). The absence of acute arterial obstruction was confirmed, indicating that RBSS can coexist with chronic hypertensive ocular disease. After discussing treatment options, the patient underwent cyclophotocoagulation, which reduced the IOP to 21 mmHg in the right eye and 27 mmHg in the left eye. With the addition of local IOP-lowering medications, brinzolamide eye drops twice daily and timolol eye drops twice daily, no recurrence was observed during subsequent follow-up visits.

**Figure 3:**
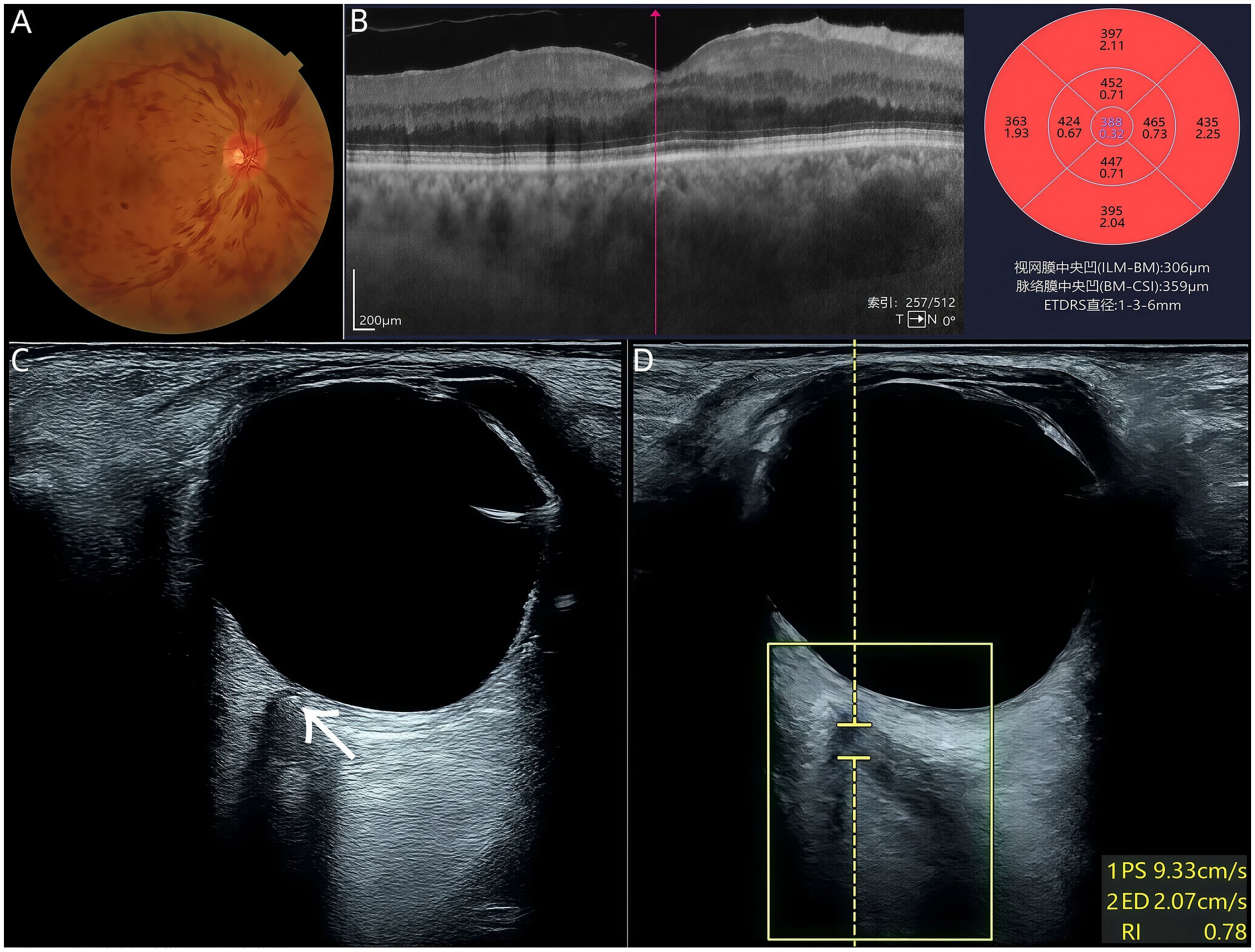
**(A)** Fundus photography of the right eye revealed mild tortuosity and venous dilation with flame-shaped hemorrhages. **(B)** OCT demonstrated diffuse retinal thickening, with central foveal thickness measuring 306 μm. **(C)** Positive RBSS in Case 3 (arrow), measuring 0.8 × 0.4 mm. **(D)** Positive RBSS in the right eye, with arterial flow showing PS 9.33 cm/s and RI 0.78.

Finally, a 67-year-old male presented with a one-month history of progressively decreasing vision, with a marked decrease in vision in his right eye over the past 2 weeks. During the initial ocular examination, his BCVA was 0.5 in the right eye and 1.0 in the left eye. His medical history revealed well-controlled hypertension for the past 2 years, with no other significant systemic conditions. Further diagnostic evaluations were conducted, including color fundus photography and OCT, which confirmed a diagnosis of central retinal vein occlusion (CRVO) in the right eye, complicated by macular edema (Figures 3A,B). Transorbital sonography revealed normal retrobulbar blood flow, a positive RBSS in the right eye (Figure 3C), and near-normal arterial perfusion (Figure 3D). These findings indicate that RBSS can occur during venous occlusion without concomitant arterial flow impairment. The patient received a single intravitreal injection of Ranibizumab, targeting the macular edema. At his one-month follow-up, the patient’s BCVA had improved to 0.8 in the affected right eye. Further monitoring and management were recommended to maintain visual stability and prevent recurrence of edema or progression of CRVO.

## Discussion

RBSS is a key feature detectable through B-mode ultrasonography; notably, all previously documented cases of RBSS have been exclusively associated with CRAO (2). However, our cases demonstrate that this sign can also occur in patients with glaucoma and CRVO, and even in healthy eyes.

These findings challenge the prevailing embolic paradigm, which posits that RBSS is a marker of acute, unilateral embolus that inevitably terminates downstream perfusion (3). In contrast, the persistence of certain RBSS in non-CRAO, its independence from flow status, and its chronic course align more closely with focal calcification of the vessel wall or adjacent neural sheath (4). The calcification model provides a coherent explanation: RBSS can remain dormant (contralateral eye, Case 1), coexist with various chronic ocular disorders (Cases 2 and 3), and occur without causing acute hemodynamic disturbances. Ultrasound findings of RBSS closely resemble the optic disc drusen (ODD) sign (5, 6), suggesting that RBSS may not fully represent emboli or embolism, and it is speculated that some RBSS is a form of local tissue calcification. Therefore, the detection of RBSS should be interpreted judiciously, integrating comprehensive clinical and hemodynamic data, rather than being automatically attributed to embolism. In some cases, RBSS may represent an incidental finding rather than a causative lesion.

Written informed consent for publication was obtained from the patient and is on file. The study was conducted in accordance with the principles of the Declaration of Helsinki.

## Data Availability

The original contributions presented in the study are included in the article/supplementary material, further inquiries can be directed to the corresponding author.
